# Wavelet scattering transform application in classification of retinal abnormalities using OCT images

**DOI:** 10.1038/s41598-023-46200-1

**Published:** 2023-11-03

**Authors:** Zahra Baharlouei, Hossein Rabbani, Gerlind Plonka

**Affiliations:** 1 Medical Image and Signal Processing Research Center, School of Advanced Technologies in Medicine, Isfahan University of Medical Sciences, Isfahan, Iran; 2 Institute for Numerical and Applied Mathematics, Georg-August-University of Goettingen, Göttingen, Germany

**Keywords:** Biomedical engineering, Medical imaging

## Abstract

To assist ophthalmologists in diagnosing retinal abnormalities, Computer Aided Diagnosis has played a significant role. In this paper, a particular Convolutional Neural Network based on Wavelet Scattering Transform (WST) is used to detect one to four retinal abnormalities from Optical Coherence Tomography (OCT) images. Predefined wavelet filters in this network decrease the computation complexity and processing time compared to deep learning methods. We use two layers of the WST network to obtain a direct and efficient model. WST generates a sparse representation of the images which is translation-invariant and stable concerning local deformations. Next, a Principal Component Analysis classifies the extracted features. We evaluate the model using four publicly available datasets to have a comprehensive comparison with the literature. The accuracies of classifying the OCT images of the OCTID dataset into two and five classes were $100\%$ and $82.5\%$, respectively. We achieved an accuracy of $96.6\%$ in detecting Diabetic Macular Edema from Normal ones using the TOPCON device-based dataset. Heidelberg and Duke datasets contain DME, Age-related Macular Degeneration, and Normal classes, in which we achieved accuracy of $97.1\%$ and $94.4\%$, respectively. A comparison of our results with the state-of-the-art models shows that our model outperforms these models for some assessments or achieves nearly the best results reported so far while having a much smaller computational complexity.

## Introduction

The retina is the innermost layer in the eye that creates vision. Various diseases have been diagnosed in this sensitive part of the eye, which affect different layers of the retina. In Diabetic Retinopathy (DR); retinal blood vessels can leak or become blocked. Several changes, such as increasing the thickness of retinal layers, are seen in this abnormality. It is a serious cumulative vascular condition that damages retinal cells with no obvious visual symptoms at first but it can progress to a widespread and severe state, and the disease’s progression can result in blindness^1^. The changes in DR involve the retinal microvasculature specifically the tight junctions of the endothelial cell wall^2^. Age-related Macular Degeneration (AMD) usually appears with thickness in the Retinal Pigment Epithelium (RPE) layer. AMD originates either from the choroid or, less frequently, from the retinal circulation. The leakage in the aberrant vessels results in fluid accumulation underneath the retina and leads to rapid visual loss^3^. AMD is categorized into three stages as early, intermediate, and late stages. Two ones are non-advanced stages with no fluid or atrophy. The advanced AMD is characterized by the advanced dry stage and advanced exudative stage^4^. Macular Hole (MH) lead to distorted or blurred vision, as well as a decrease in visual acuity. Thickened edges, fluid accumulation, and macular edema are signs of MH. An important factor in the development of MH is parafoveal vitreous detachment. Anteroposterior traction with parafoveal vitreous detachment may be involved in the onset and development of MH^5^. Central Serous Retinopathy (CSR) is an eye condition characterized by the accumulation of fluid under the retina in the central macular area. Leakage of fluid into the retina through an RPE defect is seen in CSR^6^. In this disease, dysfunctional retinal pigment epithelial cells and/or choroid lining the retina lead to the development of sub-retinal fluid^7^.

Retinal abnormalities are diagnosed through observation of the retinal images. Optical Coherence Tomography (OCT) is a widely accessible, non-invasive medical imaging technique that uses light to capture pictures at microscopic resolution from the retina^8^. Manual diagnosis of retinal abnormalities is costly and time-consuming and also requires highly trained clinicians to have precision. Early diagnosis of such pathologies can decrease the risk of vision loss and the cost of treatment. Recently, computer-aided diagnosis (CAD) in retinal OCT has been considered to assist ophthalmologists in the early detection of retinal pathologies. In this line, new machine learning and deep learning algorithms have been proposed for pre-processing, abnormality diagnosis, segmentation, and classification of OCT images^9–15^.

Deep learning-based methods have been shown to generally outperform classical machine learning methods^16^. However, there are also various disadvantages, such as the requirement for using a large training datasets, increasing complexity or processing time; and lack of interpretability^17,18^. Unclear extracted features and decision methods in the network layers may not be helpful for some clinical applications in real life^19^. Furthermore, a high-performance deep learning method that is adjusted for a specific dataset may not be appropriate for different datasets.

To overcome the mentioned shortages in deep learning architectures, a particular CNN was proposed using Wavelet Scattering Transforms (WST) in^20,21^. Since WST contains a cascade of wavelet transform convolutions and nonlinear modulus and averaging operators in each layer, it can be interpreted as a convolutional neural network. Convolution networks cascade convolutions and pooling nonlinearity, which in WST is the modulus of a complex number. The wavelet scattering network provides frequency and time resolutions. This transform preserves high-frequency information for classification, and is invariant to translations. Moreover, it is stable to small local deformations. It takes advantage of CNN while reducing its adverse properties^22^.

In this paper, we want to diagnose retinal diseases using OCT images applying WST. We do not employ any pre-processing of the data but rely on the decorrelation property of the wavelet transform. In this way, we obtain an efficient model with essentially decreased computational cost compared to deep learning models. Using only two layers of the WST network, we can already achieve comparable accuracy with the state-of-the-art methods.

To evaluate the model, we use several datasets. We get OCT images from the OCTID dataset^23^ with five classes and 572 images, to show the accuracy of this method on a small number of images and the large number of classes as was shown in^24^. We also evaluate the model using the TOPCON (which includes two classes and 57171 images)^25^, the Heidelberg (which includes three classes and 4254 images)^26^, and the Duke (which includes three classes and 3231 images)^27^ datasets to show the generalization of the method which achieves relatively good accuracy on different datasets with different properties such as technologies, the number of images and classes, and with different dimensions. Without using any pre-processing step and with the small number of layers, we propose a very efficient model to classify the OCT images, which is implementable in practice. After data processing using WST, a Principle Component Analysis (PCA) based classifier is implemented for classification. The results show that using WST, good accuracy can be achieved for the classification of OCT images with this simple architecture.

The novelty and the contribution of this work can be summarized as follows:

- For the first time, we use the WST method to detect retinal abnormalities using different OCT datasets.
- To decrease the computational complexity and increase the speed, we don’t use any pre-processing on the images. We also use only two layers of WST.
- To show the accuracy of this method on different datasets with different numbers of classes and images, and different technologies, we test the method on four well-known datasets.
- We show that this architecture can achieve an acceptable accuracy with a small amount of data which is important in medical applications.
- We reach accuracies comparable to state-of-the-art methods. In some cases, this method outperforms the others.

The rest of the paper is organized as follows: First, we have a literature review in section “Related works”. The section “Materials and Methods” introduces the datasets and describes the method. In section “Results” the experimental results are presented. In the section “Discussion”, we summarize the results and analyze them. Section “Conclusion” summarizes the article.

## Related works

The results of previous classification methods in the literature differ concerning dataset properties (such as the contrast of images, imaging system, noise level, size of dataset), the network depth, the generality of the algorithm, computational complexity, and processing time. Therefore, the methods cannot be easily compared^19^. For example^28^, achieved an accuracy of $88.4\%$ using 2000 images from the EyePACS dataset, while^18^ reported an accuracy of $97.93\%$, using a more complex network and 35,126 images from the same dataset. Authors in^29^, used a four layers Convolutional Neural Network (CNN), and reported accuracies of $87.83\%$ using pre-processing and $81.8\%$ without it.

Some papers focus on diagnosing only one particular disease. In He et al.^30^, AMD was diagnosed from Normal cases using ResNet-50. The AUC of 0.99, Sensitivity of $95.02\%$, and Specificity of 95.02 were the reported results. Dry AMD (drusen) versus wet AMD was diagnosed from OCT images using FPN-VGG-16 which lead to $93.4\%$ accuracy^31^. In An et al.^32^, AMD with fluid versus AMD without fluid using VGG-16 achieved to the accuracy of $95.1\%$.

Thomas et al.^33^ used Recurrent Neural Network (RNN) for the classification of AMD from Normal ones. Many articles have addressed DR detection, e.g.^34–41^. Several papers used Deep CNN (DCNN) method on various datasets, e.g.^35–38^, to detect DR. The obtained accuracy differs from $82.1$ to $99.7\%$.

Some other papers tried to diagnose two and more diseases using different methods and datasets. Rasti et al.^43^ recognized AMD, DME, and Normal cases with an accuracy of $98.14\%$, using a multi-scale convolutional mixture of experts, while^44^ diagnosed the same classes with an accuracy of $92.06\%$, using surrogate CNN. Using a wavelet-based CNN model, an accuracy of $98.67\%$ was achieved for the three-class classification task in Kafieh et al.^25^. In Elmoufidi et al.^45^, Different stages of DR were detected using CNN.

In addition to OCT images, some datasets acquired by other imaging technologies such as Fundus and OCT Angiography (OCTA) are used in the papers. Fundus is preferred for vascular diseases^46–49^. Hacisoftaoglu^47^ using smartphone based methods on some datasets with Fundus images achieved to $98.6\%$ of accuracy. Using DCNN, 10-fold cross-validation, an accuracy of $99.28\%$ was achieved in Shankar et al.^48^. Some researchers evaluated their works using both OCT and Fundus images, e.g.^2,49^. OCTA has recently attracted the attention of researchers. It’s a non-invasive imaging technique used in ophthalmology to visualize the blood vessels in the retina and choroid (the vascular layer behind the retina). Different studies of classification and segmentations are performed on such images, e.g.^50–53^.

A review of the retinal diseases classification results shows that deep learning based methods mostly have higher performance than basic machine learning ones. Basic machine learning methods usually have higher rates. In Sandhu et al.^54^, the authors tried to reduce the image dimensions and improve the classification performance, using the feature bagging technique. They achieved an accuracy of $80\%$ with low computational time. In Somasundaram and Ali^55^, by extracting wavelet features and using four classification methods, $82\%$ accuracy was obtained. In some basic machine learning models, high accuracy was achieved using special pre-processing techniques. For example, in Ali^56^, a novel pre-processing method was proposed, different features were extracted, and five classification methods were implemented to achieve an average accuracy of $98.83\%$. Compared with^54^, improving the accuracy in Ali^56^ was in return for increasing the processing time. Most CNN-based methods and specifically, DCNN models, achieved higher accuracy than others. For example^38,48,57^, achieved the best accuracy of $99.1\%$, $99.28\%$, and $99.73\%$, respectively in detecting DR grades using DCNN models.

## Materials and method

**Figure 1:**
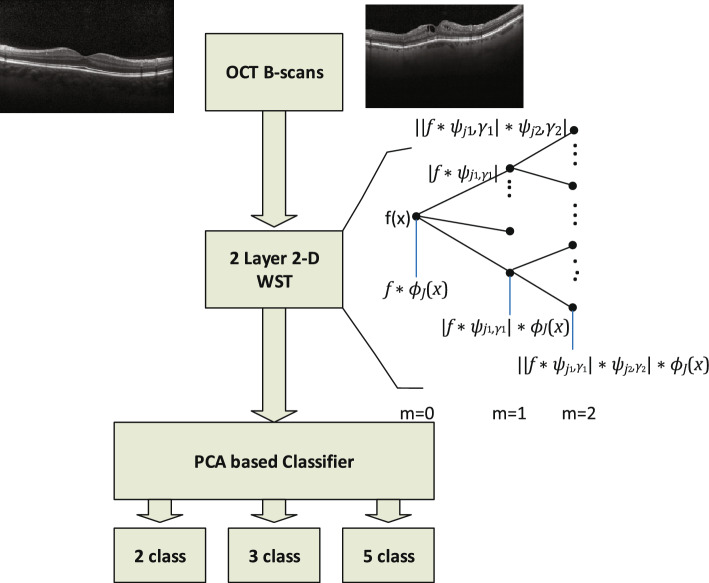
Block diagram of the model.

In this work, we aim to diagnose retina diseases from OCT images. We use the Wavelet Scattering Transform (WST) to access a sparse representation of images. Next, we employ a PCA-based classifier to categorize the retina diseases into different classes. We test our model on different OCT datasets to verify the accuracy of the model. We use the OCTID dataset to show the relatively good accuracy of the model to detect diseases from a large number of classes and a small amount of training data. Finally, we also use some well-known datasets involving a different number of images in 2 or 3 classes to compare the accuracy with state-of-the-art models in the literature. The block diagram of the architecture is shown in Fig. 1.

In the rest of this section, we explain the used datasets, the method, and the classification in more detail.

### OCT datasets

In this work, four open-access datasets of OCT images are used. In the following, we describe the details of the OCTID^23^, TOPCON^25^, Duke^27^, and Heidelberg^26^ datasets.

The OCTID dataset includes 572 OCT images that are categorized into five classes Normal, CSR, MH, AMD, and DR. Images have $586 \times 879$ pixel resolution and 2 mm scan length, which are obtained from a raster scan protocol using a Cirrus HD-OCT machine^23^.

The TOPCON dataset includes 57171 B-scans of DME and Normal images with $650 \times 512$ resolution obtained from the Topcon 1000 device in the Ophthalmology Dept., Feiz Hospital, Isfahan, Iran.

The Duke-Harvard-Michigan Heidelberg dataset contains 45 cases of AMD, DME, and Normal with a total of 3231 OCT images, which have $496 \times 1024$ resolution.

The dataset from the Heidelberg device was acquired at Noor Eye hospital in Tehran containing 50 Normal and DME, and 48 AMD cases with a total of 4254 OCT images. The resolution of images is $512 \times 496$.

**Figure 2:**
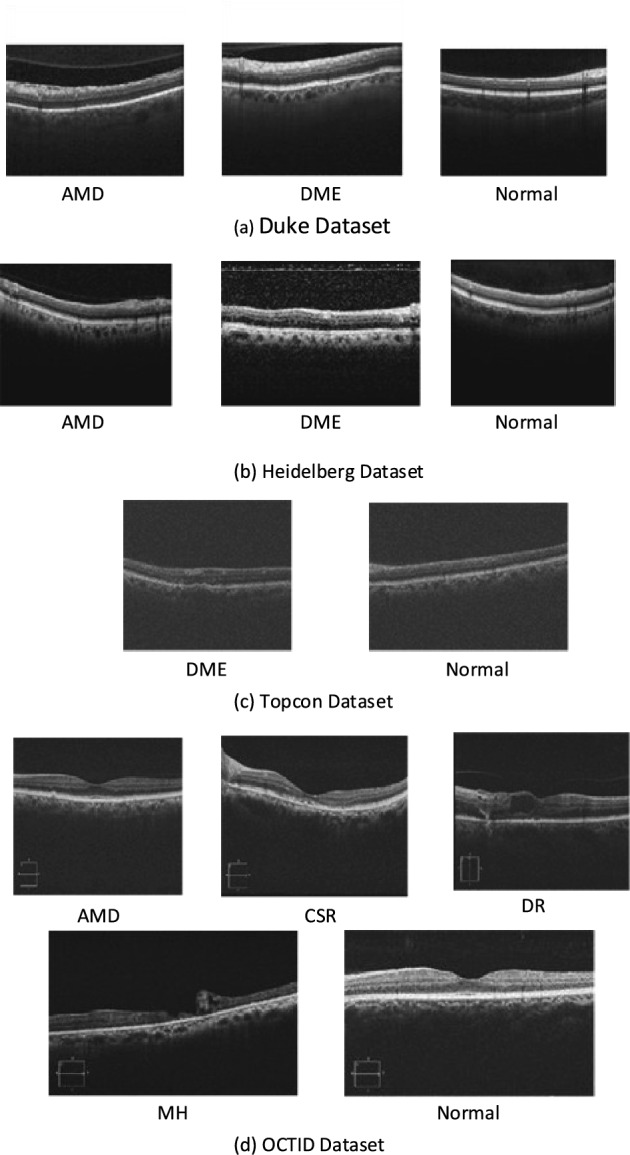
A sample of the OCT images in the datasets.

A sample of the images in each class of these datasets is presented in Fig. 2 and the properties of the used datasets in this work are listed in Table 1.

**Table 1:** OCT datasets used in this work.

Dataset	No. Images	Access	Resolution	Classes
OCTID^23^	572	OA	$586 \times 879$	Normal (206), CSR (102), MH (102), AMD (55), and DR (107)
Duke^27^	3231	OA	$496 \times 1024$	Normal (1407), DME (1101), and AMD (723)
Heidelberg^26^	4254	OA	$512 \times 496$	Normal (1585), DME (1104), and AMD (1565)
TOPCON^25^	57171	OA	$650 \times 512$	Normal (33313) and DME (23858)

### Method

#### Wavelet scattering transform

We want to use a model with low computational cost and a high classification rate to be implementable in practice for medical tasks. In this model, we use WST to extract the important image features. Unlike deep learning models, the WST can be easily interpreted. The scattering coefficients at each scale and orientation capture different levels of signal information which are crucial for better classification. The WST is designed to be invariant to deformations, rotations, and translations, making it robust to variations in the input signal. This is particularly important in medical imaging applications where the position and orientation of the part being imaged can vary. Moreover, it preserves high-frequency information.

The WST requires fewer training examples than deep learning methods, making it a good choice for applications where labeled data is limited. We need a much smaller amount of training data to achieve clear discrimination of up to five classes.

This method is computationally efficient that can handle large volumes of data. This makes it a good choice for applications where real-time processing is required. Our results show that only two layers in this network are sufficient to achieve very good classification results.

We feed 2-D OCT images, without any pre-processing, to a WST architecture. After transferring the OCT images to the sparse representation, a PCA-based classifier categorizes the retina diseases into different classes

In the following we briefly summarize the WST approach in the continuous setting.

Let $f({{\textbf{x}}})$ with ${{\textbf{x}}} = (x_{1}, x_{2})^{T}$ be the two-dimensional signal on a rectangular (image) domain $\Omega \subset {{\mathbb {R}}}^{2}$. In the first step, the image *f* is filtered by applying convolutions with the scaled Gaussian (low-pass) function $\phi$ and a scaled and rotated (band-pass) wavelet function $\psi$. Then we take the modulus of these convolutions and apply a localized averaging by convolution with the scaled Gaussian $\phi$. As in Bruna and Mallat^22^, let

$$\begin{aligned} \phi _J({{\textbf{x}}})=2^{-2J}\phi \left( 2^{-J}{{\textbf{x}}} \right) , \end{aligned}$$

where $\phi ({{\textbf{x}}}) := \frac{1}{2\pi \sigma ^{2}} \exp (-|{{\textbf{x}}}|^{2}/2 \sigma ^{2})$ is the two-dimensional Gaussian window function with $\sigma = 0.85$. Then

$$\begin{aligned} ({{\mathscr {S}}}_{0,J} f)({{\textbf{x}}}):= (f * \phi _{J})({{\textbf{x}}}) = \int _{\Omega } f({{\textbf{y}}}) \, \phi _{J} ({{\textbf{x}}}-{{\textbf{y}}}) \, d{{\textbf{y}}} \end{aligned}$$

is the zeroth order scattering coefficient representing the low-pass part of *f*. Next, we consider the two-dimensional Morlet wavelet

$$\begin{aligned} \psi ({{\textbf{x}}}):= c_{1} ( {\mathrm e}^{3\pi {\mathrm i}{\textbf{x}}/4} - c_{2}) \phi ({{\textbf{x}}}), \end{aligned}$$

where $c_{1}$ is a normalization factor and $c_{2}$ is chosen such that $\int _{{{\mathbb {R}}}^{2}} \psi ({{\textbf{x}}}) \, d {{\textbf{x}}} =0$. In other words, $\psi ({{\textbf{x}}})$ is the difference between a plane wave and a constant, localized by the Gaussian window $\phi ({{\textbf{x}}})$, and can be interpreted as a band-pass filter.

Further, let $\Gamma :=\{0, \frac{\pi }{r}, \frac{2\pi }{r}, \ldots , \frac{(r-1)\pi }{r}\}$ be a fixed set of *r* equidistant rotation angles in $[0, \pi )$ where we usually set $r=12$ in our experiments. Then the scaled and rotated wavelet functions are determined by

$$\begin{aligned} \psi _{j,\gamma }({{\textbf{x}}}):= 2^{-2j} \, \psi (2^{-j} {\textbf{R}}_{\gamma } {{\textbf{x}}}), \qquad j=0, \ldots , J-1, \, \gamma \in \Gamma , \end{aligned}$$

where $${{\textbf{R}}}_{\gamma } = \left( \begin{array}{cc} \cos \gamma &{} \sin \gamma \\ - \sin \gamma &{} \cos \gamma \end{array} \right)$$ denotes the rotation matrix corresponding to $\gamma \in \Gamma$. The vector of scattering coefficients of the first order is now given by

$$\begin{aligned} {{\mathscr {S}}}_{1,J}f ({{\textbf{x}}}):= \{ (|f * \psi _{j_{1},\gamma _{1}}| * \phi _{J})({{\textbf{x}}}): \, j_{1}=0, \ldots , J-1, \, \gamma _{1} \in \Gamma \}. \end{aligned}$$

Indeed, the $L^{1}({{\mathbb {R}}}^{2})$-norm $|f * \psi _{j_{1},\gamma _{1}}|_{1} = \int _{\Omega } |(f * \psi _{j_{1},\gamma _{1}})({{\textbf{x}}})| \, d{{\textbf{x}}}$ is obviously translation-invariant. Employing the convolution with a wide Gaussian window $\phi _{J}$ gives a similar result, i.e., we have almost translation invariance, i.e., we have ${{\mathscr {S}}}_{1,J} f({{\textbf{x}}} + {{\varvec{\tau }}}) \approx {{\mathscr {S}}}_{1,J} f({{\textbf{x}}})$ if the components of ${\varvec{\tau }}$ are small enough. The scattering coefficients of the first order are equivalent to the feature vector obtained in the Scale-Invariant Feature Transform (SIFT), a locally invariant image descriptor proposed in Lowe^42^. The convolution of $|f * \psi _{j_{1},\gamma _{1}}({{\textbf{x}}}) |$ with the Gaussian window $\phi ({{\textbf{x}}})$ is a low-pass filtering procedure that causes an information loss. To achieve improved high-frequency information, the vector of scattering coefficients of the second order is computed as

$$\begin{aligned} {{\mathscr {S}}}_{2,J}f ({{\textbf{x}}}):= \{ (||f * \psi _{j_{1},\gamma _{1}}| * \psi _{j_{2},\gamma _{2}}| * \phi _{J})({{\textbf{x}}}): \, j_{1}=0, \ldots , J-1, \, j_{2} = j_{1}, \ldots , J-1, \, \gamma _{1}, \gamma _{2} \in \Gamma \}. \end{aligned}$$

**Figure 3:**
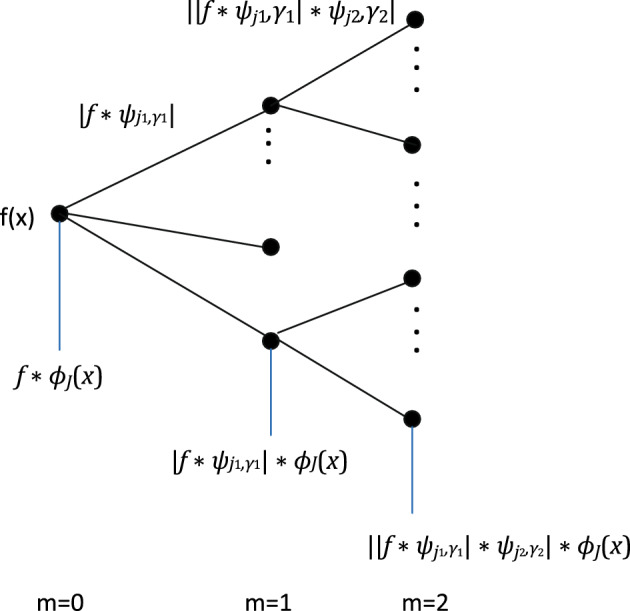
The wavelet scattering network with two layers.

More translation-invariant scattering coefficients can be computed by iterating this procedure, and the energy of the image signal *f* is propagated across the scattering coefficients. As has been shown in Bruna and Mallat^60^, the scattering coefficients of order 0 to 2 in

$$\begin{aligned} {{\mathscr {S}}}_{0,J}f ({{\textbf{x}}}), \, {{\mathscr {S}}}_{1,J}f ({\textbf{x}}), \, {{\mathscr {S}}}_{2,J}f ({{\textbf{x}}}) \end{aligned}$$

contain usually already more than 98 % of the energy of *f*. Thus we use only the coefficients in layers 0, 1, 2, which reduces the computational complexity significantly. Figure 3 shows the WST with $m=2$ used in this work. Observe that in the considered continuous setting the image *f* as well as all scattering coefficients are still functions on $\Omega$. We set the dimension of the scaling filter, called invariant scale, equal to the minimum dimension of the images for each dataset used in this paper. In practice, we have a given discrete image ${{\textbf{f}}}$ with *N* pixels and the convolutions have to be discretized. The total number of scattering coefficients in ${{\mathscr {S}}}_{1,J}$ is *Jr* and the number of scattering coefficients in ${{\mathscr {S}}}_{2,J}$ is $r^{2}\frac{J(J-1)}{2}$, where *r* is the number of considered angles. These functions are uniformly sampled with grid size $2^{J}$ such that each discretized scattering coefficient has $2^{-2J}N$ coefficients, where *N* is the number of pixels of the image ${{\textbf{f}}}$. Together, the total number of the discrete feature vectors $S_{J} {{\textbf{f}}}$ of ${{\textbf{f}}}$ (consisting of components of all feature coefficients of order 0, 1, and 2) is then $N_{J}:=(1+rJ+ r^{2}\frac{J(J-1)}{2}) 2^{-2J} N$.

#### Classifier

We employ a classifier based on PCA applied to a suitable affine space, as proposed in Bruna and Mallat^22^. The classification algorithm directly employs the scattering coefficient vectors $S_{J} {\textbf{f}}$. Assume that we have computed a complete vector of scattering coefficients of length $N_{J}$ that contains the scattering coefficients of ${{\textbf{f}}}$ of order $0, \, 1$, and 2 at subsampled positions. Each signal class is represented by a random vector ${{\textbf{f}}}_{k}$, and the realizations of this random vector are the images ${{\textbf{f}}}$ in this class. Let $E(S_{J}{\textbf{f}}_{k})$ denote the expected scattering coefficient vector of length $N_{J}$ of images ${{\textbf{f}}}$ in class *k*. Further, let ${{\textbf{V}}}_{d,k}$ be the rank-*d* approximation of the covariance matrix of $S_{J}{{\textbf{f}}}_{k}$ of size $N_{J} \times N_{J}$ built by the eigenvectors of the covariance matrix corresponding to the largest *d* eigenvalues. In our experiments, we have used $d=30$. We obtain the affine approximation space

$$\begin{aligned} {{\textbf{A}}}_{d,k} = E(S_{J}{{\textbf{f}}}_{k}) + {{\textbf{V}}}_{d,k}, \end{aligned}$$

see also^22^. Having found this affine space, the classifier associates an image ${{\textbf{f}}}$ to the class *k* (among *K* classes) if

$$\begin{aligned} k({{\textbf{f}}}) = \text {argmin}_{1 \le k' \le K} \Vert S_{J}{\textbf{f}} - P_{{{\textbf{A}}}_{d,k}} (S_{J}{{\textbf{f}}})\Vert _{2}, \end{aligned}$$

where $P_{{{\textbf{A}}}_{d,k}}$ denotes the projection onto the affine space ${{\textbf{A}}}_{d,k}$.

The computational effort for the classification is governed by the required singular value decomposition of the covariance matrix of $S_{J}{{\textbf{f}}}_{k}$ with $O(N_J^3)$ floating point operations.

## Results

To assess the model, we classified the OCT images of the OCTID, TOPCON, Duke, and Heidelberg datasets. These datasets differ in technologies, the number of images and their dimensions, and also the number of classes. The wavelet scattering features are extracted, and a PCA-based classifier is used to diagnose the retinal abnormalities. In this work, a wavelet scattering transform in Matlab was implemented. As mentioned in the Method Section, the energy of signals is significantly decreased as the layers are increased. Using two layers of wavelet filter banks is sufficient for classifying OCT images. For each wavelet filter, different rotations from 6 to 12 in $[0,\pi ]$ were considered. The best results were related to 12 rotations for all datasets except for OCTID, in which increasing the rotations number did not have any effect on the results. The spatial support in the row and column dimensions of the scaling filter was considered as half of the minimum dimension of the images for each dataset. To train the network, we used $80\%$ of the data, and the rest of $20\%$ was used to test.

**Figure 4:**
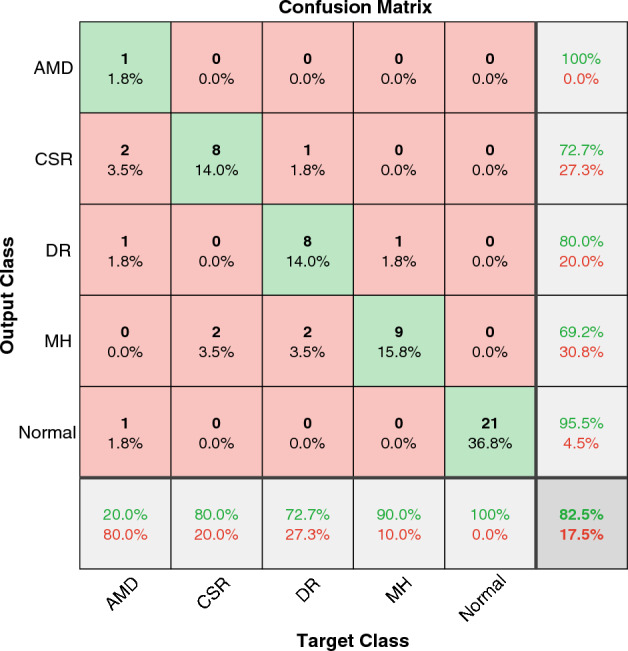
The confusion matrix of WST on OCTID dataset for diagnosing five classes of OCT images.

We tested our model to investigate the accuracy of diagnosing five categories in the OCTID dataset. The result is shown in Fig. 4. The accuracy of this classification is $82.5\%$. Only one work in the literature reported the classification results for five classes in OCTID^61^. In Mishra et al.^61^, the accuracy of $\%93.12 (+/- 8.59)$ was reported using a CNN model. The model includes 13 convolution layers, 4 Maxpool layers, three fully connected layers, an attention module, and reshape, normalization, flatten softmax, and lost steps. Comparing the process steps and network layers in Mishra et al.^61^ with our model shows the trade-off between computational complexity and processing time with accuracy. This is other than the shortages in using black-box CNN models.

**Figure 5:**
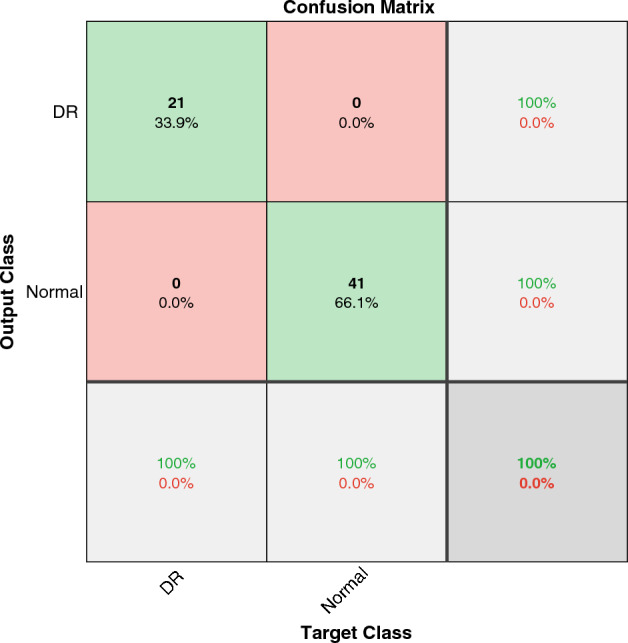
The confusion matrix of WST on OCTID dataset for diagnosing DR from Normal cases.

Most of the classification works on the OCTID dataset investigated the detection accuracy for two classes, one abnormality from Normal ones. We examined our model for detecting DR pathology, which is one of the most common diseases in diabetic patients. Figure 5 shows that our method achieved $100\%$ of accuracy for DR detection. Table 2 compares our result with other works in detecting DR. As seen in the table, this model outperforms other state-of-the-art models.

**Table 2:** Comparing DR detection accuracy in different works.

Paper	Year	Method	Dataset	Accuracy
^47^	2020	Deep learning, Smartphone based method	EyePACS, IDRiD, MESSIDOR, MESSIDOR-2	98.6%
^48^	2020	DCNN, classification based on 10-fold cross validation	MESSIDOR	99.28%
^62^	2021	Transfer learning on Inception-ResNet-V2	Kaggle dataset, MESSIDOR	Kaggle dataset: 72.33%, MESSIDOR: 82.18%
^63^	2021	Hybrid Inductive Machine Learning	CHASE	96.62%
^39^	2022	Graph-CNN	OCTID	98%
^40^	2023	2-stage noninvasive framework	SD-OCT	93.8%
^41^	2023	CNN	DRIL	88.3%
This paper		WST	OCTID	100%

**Figure 6:**
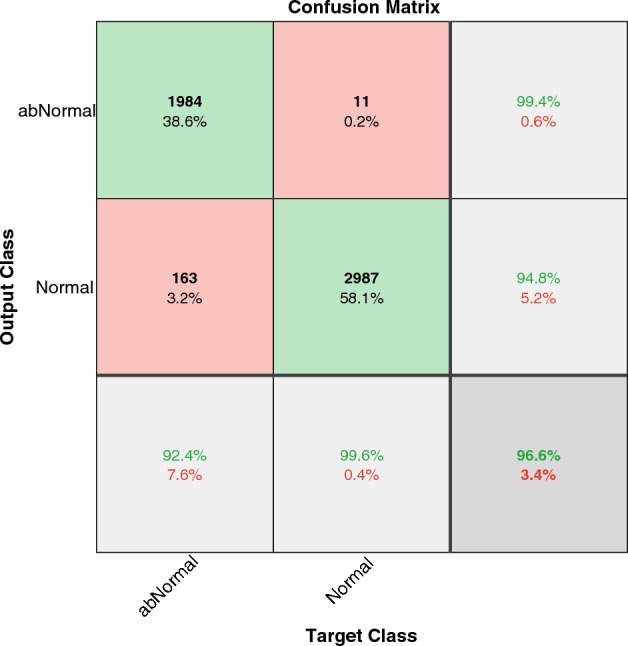
The confusion matrix of WST on the TOPCON dataset for diagnosing DME from Normal cases.

Next, we tested our model on the TOPCON dataset. An accuracy of $96.6\%$ was achieved in detecting DME from normal ones, as seen in Fig. 6. We listed the best results that have been reported in the literature in Table 3, to compare the results with other works. As seen in the table, most CNN-based works achieved a higher accuracy. The WST-based model in this paper can achieve accuracy close to the complicated architecture of CNN-based models using a simple architecture.

**Table 3:** Comparing DME detection accuracy in different works.

Paper	Year	Method	Dataset	Accuracy (%)
^67^	2011	Multiscale LBP, classification based on 10-fold cross validation	TOPCON	95.9
^27^	2014	Multiscale HOG	TOPCON	96
^64^	2017	CNN (VGG-16)	SERI	87.5
^65^	2018	CNN (VGG-16), classification based on 32-fold cross validation	SERI	93.75
^25^	2018	CNN (WCNN1), classification based on 5-fold cross validation	TOPCON	99.3
^66^	2022	CNN (DeepOCT)	ZhangLab	99.2
This paper		WST	TOPCON	96.6

**Figure 7:**
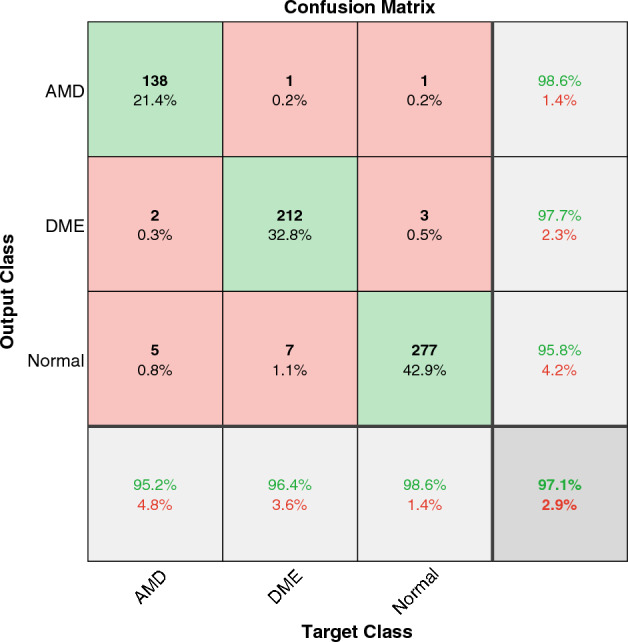
The confusion matrix of WST on the Duke dataset.

**Figure 8:**
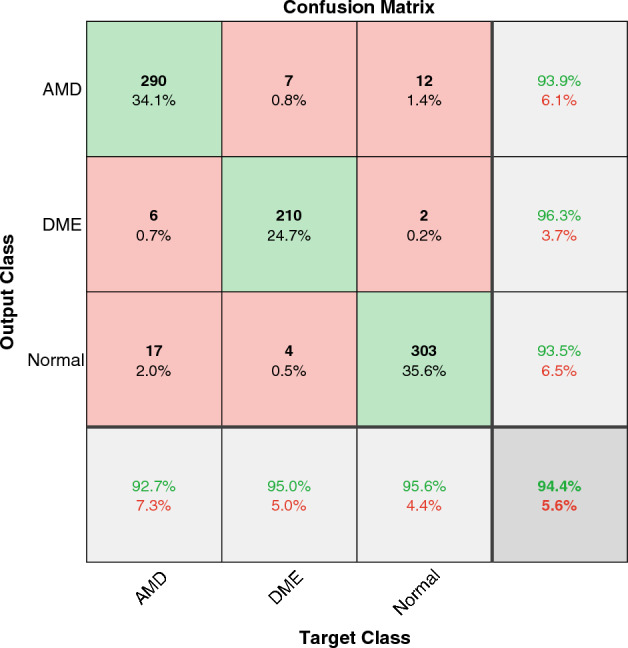
The confusion matrix of WST on the Heidelberg dataset.

To compare the performance of the work with the research on other well-known datasets, we tested our model on the Duke and Heidelberg datasets to diagnose DME and AMD from Normal ones. We achieved the accuracy of $97.1\%$ and $94.4\%$, respectively. The results are shown in Figs. 7 and 8.

The best results reported in the literature on Duke and Heidelberg datasets are compared in Tables 4 and 5. The results show that we achieved the best accuracy in classifying on the Duke dataset. Since most of the works on the Duke dataset used k-fold cross-validation, we also implemented 10-fold validation to have a fair comparison. We achieved $96.7\%$ of accuracy which is the best result reported in the literature and equal to the one in Thomas et al.^33^. The classification accuracy of this work on the Heidelberg dataset is close to the best results in the literature but less than some. An overall view of the results on different datasets shows that this model achieves similarly good classification results as the other state-of-the-art models, specifically the CNN-based ones.

**Table 4:** Comparing DME, AMD, and Normal detection accuracy in different works using the Duke dataset.

Paper	Year	Method	Classes	Accuracy
^33^	2021	Multipath CNN, classification based on 10-fold cross validation	AMD, Normal	96.7%
^68^	2021	Statistical method	AMD, Normal	96.6%
^70^	2021	DL (VGG-16)	AMD,DME, Normal	94.2%
^69^	2022	Classical ML (n-gram), classification based on 10-fold cross validation	AMD, DME, Normal	AMD:86.7% DME:93.3% Normal: 93.3%
This paper		WST	AMD DME Normal	97.1%
		WST, classification based on 10-fold cross validation		96.7%

**Table 5:** Comparing DME, AMD, and Normal detection accuracy in different works using the Heidelberg dataset.

Paper	Year	Method	Classes	Accuracy (%)
^43^	2018	CNN (MCME), classification based on 5-fold cross validation	AMD, DME, Normal	99.01
^73^	2019	DL CliqueNet based	AMD, DME, Normal	98
^72^	2021	CNN	AMD, DME, Normal	93.87
^33^	2021	Multipath CNN, classification based on 10-fold cross validation	AMD, Normal	98.9
^71^	2022	DL(fine-tuned)	AMD, DME, Normal	96.5
This paper		WST	AMD, DME, Normal	94.4

## Discussion

In this article, we used the WST-based method to diagnose retinal diseases from OCT images. We achieved different accuracies for the four databases used. Comparing the accuracy obtained in this method with other methods in Tables 2, 3, 4 and 5 shows that this method is generally comparable with state-of-the-art and highly accurate methods. As mentioned, the presented results are using two layers of the WST. We have shown the effect of using fewer layers on the results in Supplementary Appendix 1. In the appendix, we have also discussed the cause of failure cases in the classification.

Among the advantages of this method over deep learning methods is short processing time. The computational cost for the WST only depends on the input size of the image, the chosen predefined scale $2^J$ and the number of angles *r*, and can be given as $O(N_J \log (N))$ for an image with *N* pixels. This means, the effort to perform the WST is even smaller than the needed cost to compute the low rank approximation of the correlation matrix of size $N_{J} \times N_{J}$ for classification.

In Table 6 we report all the obtained accuracies in this paper. Considering accuracy, our method outperforms previous research in DR detection using the OCTID dataset (with a very small amount of data) and on the Duke dataset. In other cases, the accuracy of our method is not much different from the best results obtained.

We also calculated AUC (Area under the ROC Curve). According to Table 6, our method has the best AUC on the Duke dataset, but this result is lower compared to previous research reports, which mostly reached an AUC above 0.9.

Using ANOVA statistical testing, we calculated the *P*-value for the experiments. The best results were achieved in the experiment performed on the OCTID dataset with five classes and on the TOPCON dataset, as seen in the table.

**Table 6:** The experimental results of using the WST on four OCT datasets.

Dataset	Numer of Images	Number of classes	Acc	AUC	P-value
OCTID	572	5	$82.5\%$	0.87	0.0109
OCTID	313	2	$100\%$	0.78	0.333
Duke	3231	3	$97.1\%$	0.88	0.2385
Heidelberg	4254	3	$94.4\%$	0.82	0.94
Topcon	57171	2	$96.6\%$	0.68	0.0344

## Conclusions

Various retinal diseases can be diagnosed using OCT images. To overcome some shortages in manual diagnosing, such as mistakes and costs, computer-aided manners have been considered today. Various classical machine learning and deep learning methods have been proposed in this field. Although deep learning techniques, specifically CNN-based methods, can achieve high accuracies in detecting different abnormalities, some shortages make them often impractical. Application problems in practice include the high computation complexity, long processing time, requirement of large datasets, and unclear interpretability.

In this paper, we implemented the wavelet scattering network to diagnose retinal abnormalities using OCT images. This transformation overcomes some mentioned shortages of CNN methods. In particular, the CNN of the WST is based on predefined wavelet filters. Employing only two layers of the WST, we achieved an efficient model with low computational complexity.

This is the first time that WST was used on OCT images. In previous research, WST-based methods have been proposed for the classification of EEG and ECG signals, and in most cases, good results have been achieved compared to other methods. In this article, using this method and without pre-processing, we categorized retinal diseases using several OCT databases to obtain an evaluation of the different numbers of image classes, technologies, and sizes of images. We performed a comprehensive assessment and comparison of the method.

The accuracies of classifying the OCT images of the OCTID dataset into five and two classes were $82.5\%$ and $100\%$, respectively. We achieved an accuracy of $96.6\%$ in diagnosing DME from Normal ones using the TOPCON device-based dataset. The Heidelberg and the Duke datasets contain DME, AMD, and Normal classes, where we achieved $97.1\%$ and $94.4\%$, respectively.

Comparing our results with the state-of-the-art models in the literature shows that this model outperforms the compared models in detecting DR in the OCTID and the Duke dataset with three classes. In other cases, our results are comparable with other works, specifically with CNN-based techniques. An acceptable decrease in accuracy of some assessments was seen comparing the best results that have been reported in the literature, in return for an essential decrease of the computational complexity and processing time which are essential factors in practice.

Although the classification results with this method are generally good, it still needs to be improved. In future works, we aim to upgrade the method by finding more proper wavelet filters that are particularly adapted to the special features of OCT images and which can increase the performance of diagnosing retinal disease. We also examine the effectiveness of this method to detect real samples.

### Supplementary Information

Supplementary Information.

## Data Availability

The authors declare that the data supporting the findings of this study are available at the links below: The OCTID dataset is available at: https://borealisdata.ca/dataverse/OCTID?q=&types=datasets&sort=dateSort&order=desc&page=1. The TOPCON dataset is available at: https://misp.mui.ac.ir/en/topcon-3d-oct-diabetic-data-denoising-0. The Douck dataset is available at: https://people.duke.edu/$\sim$sf59/Srinivasan_BOE_2014_dataset.htm. The Heidelberg dataset is available at: https://misp.mui.ac.ir/en/dataset-oct-classification-50-normal-48-amd-50-dme-0.
