# Supplementary material for: Wavelet scattering transform application in classification of retinal abnormalities using OCT images
Source: Sci Rep. 2023 Nov 3;13:19013. doi: 10.1038/s41598-023-46200-1 (PMC10624695; doi:10.1038/s41598-023-46200-1)
Supplement: Supplementary file 1 — Supplementary Information. [file 41598_2023_46200_MOESM1_ESM.docx]

**Supplementary Information**

**Title of article:** Wavelet Scattering Transform Application in Classification of Retinal Abnormalities using OCT Images

**Authors:** Zahra Baharlouei, Hossein Rabbani, and Gerlind Plonka

**Corresponding author:** Hossein Rabbani, Medical Image & Signal Processing Research Center, School of Advanced Technologies in Medicine, Isfahan University of Medical Sciences, Isfahan, Iran, 8174673461, **email:** h_rabbani@med.mui.ac.ir

**Appendix 1**

We examined the effect of decreasing the layers in the WST architecture on the accuracy. Using m=1 for classification on the OCTID dataset, we achieved an accuracy of 40.4% for five classes. Comparing 82.5%, which was achieved by using 2 layers (m=2), the accuracy decreased 51%. The confusion matrix is shown in Fig. S1. This result shows the effect of using the second layer on the accuracy of the proposed model.

However, even using more layers, this model has errors in detection as we saw in this paper using two layers. There are many possible reasons for the failure detection cases. For example, we focus on one case and discuss it. In the OCTID dataset, according to Figure 4, the highest detection error occurred in the AMD class. One of the reasons is that in this dataset, the number of images of this class is much less than other classes. The dataset is unbalanced, as other classes have more images than AMD class. This could cause the classifier to be biased towards the majority classes and perform poorly on the minority class (AMD).

Another reason is that some abnormalities have close symptoms that are difficult to recognize and cause errors in diagnosis. In Fig. S2 we show the test images of AMD class and determine in which category they are diagnosed. In the figure, for example the one that detected as Normal is very similar to normal cases. Maybe the AMD was in the early stages. In other cases, it seems that the lack of data balance in model training has caused errors.

**Figure S1.** The confusion matrix of WST using one layer of filter banks on OCTID dataset for diagnosing five classes of OCT images

**Figure S2. AMD class test images from the OCTID dataset and the category in which they are detected.**
